# Influence of obesity and insulin resistance with hepatic steatosis on the human plasma lipidome

**DOI:** 10.1016/j.jlr.2025.100969

**Published:** 2025-12-26

**Authors:** Max C. Petersen, Gordon I. Smith, Aaron M. Armando, Xiong Su, Oswald Quehenberger, Edward A. Dennis, Samuel Klein

**Affiliations:** 1 Division of Endocrinology, Metabolism, & Lipid Research, Washington University School of Medicine, St. Louis, MO, USA; 2 Center for Human Nutrition, Washington University School of Medicine, St. Louis, MO, USA; 3 Department of Pharmacology, University of California, San Diego, CA, USA; 4 Department of Biochemistry and Biophysics, Suzhou Key Laboratory of Systems Biomedicine, School of Life Sciences, Suzhou Medical College of Soochow University, Suzhou, China; 5 Department of Chemistry and Biochemistry, University of California, San Diego, CA, USA

**Keywords:** obesity, insulin resistance, lipidomics, complex lipids, eicosanoids

## Abstract

Insulin resistance accompanied by hepatic steatosis is a common complication of obesity. In an effort to identify plasma lipids that could be biomarkers or causes of insulin resistance with steatosis in people with obesity, we evaluated the plasma lipidome in three distinct groups separated by adiposity, hepatic steatosis, and insulin sensitivity, assessed by using the hyperinsulinemic-euglycemic clamp procedure: *i)* insulin-sensitive lean (ISL, n = 13); *ii)* insulin-sensitive obese (ISO, n = 14); and *iii)* insulin-resistant obese with hepatic steatosis (IROS, n = 13). We evaluated 759 complex lipid species in 16 subclasses (including phospholipids, glycerolipids, sphingolipids, acylcarnitines, and cholesteryl esters) and 84 eicosanoids in fasting plasma samples. Total abundances of each lipid subclass (sum of species) in the ISO group were not different from values in the ISL group, whereas phosphatidylethanolamines, triglycerides, and diacylglycerols were more abundant in the IROS than in the ISO group. The abundances of only 5 individual complex lipid species were different between the ISL and ISO groups, whereas the abundances of 23 lipids were different between the ISO and IROS groups. More complex lipids were associated with insulin sensitivity (n = 124) than obesity per se (n = 7). In contrast, plasma eicosanoids were not different between the ISO and IROS groups but were greater in both groups with obesity than in the ISL group. We conclude that insulin resistance with hepatic steatosis is associated with alterations in the plasma complex lipidome, independent of adiposity, in people with obesity, whereas adiposity has a greater impact than insulin resistance on plasma eicosanoid concentrations.

Multi-organ insulin resistance is a common metabolic abnormality associated with obesity and is a key factor in the pathogenesis of atherogenic dyslipidemia (i.e., increased triglyceride, decreased HDL-cholesterol and increased small dense LDL-cholesterol plasma concentrations), prediabetes/type 2 diabetes, and hepatic steatosis (1). However, triglycerides and cholesterol represent only a small fraction of the human plasma lipidome, which includes hundreds of physiologically and potentially clinically important lipid species that can be reliably measured (2, 3, 4). Plasma lipids can be divided into six main families: *i)* fatty acyls (saturated, monounsaturated, and polyunsaturated fatty acids including eicosanoids derived from arachidonic acid; *ii)* glycerolipids; *iii)* glycerophospholipids; *iv)* sphingolipids; *v)* sterols; and *vi)* prenols (3). Results from previous studies suggest several lipids regulate organ system function by providing substrates for oxidative metabolism and by influencing cellular signaling pathways and inflammation (4, 5, 6, 7). Accordingly, rigorous characterization of the human plasma lipidome could provide new insights into the relationships between specific lipids and metabolic function in people with obesity.

The purpose of the present study was to provide a comprehensive assessment of the plasma lipidome (assessed by using ultra-high performance liquid chromatography-tandem mass spectrometry [UPLC-MS/MS]) in three distinct groups of people characterized by body mass index, oral glucose tolerance, insulin sensitivity (assessed by using the hyperinsulinemic–euglycemic clamp procedure [HECP]) and intrahepatic triglyceride (IHTG) content (assessed by using magnetic resonance imaging) as: *i)* insulin-sensitive lean (ISL), defined as BMI <25.0 kg/m^2^ with normal oral glucose tolerance, insulin sensitivity and IHTG content; *ii)* insulin-sensitive obese (ISO), defined as BMI ≥30.0 kg/m^2^ with normal oral glucose tolerance, insulin sensitivity and IHTG content; and *iii)* insulin-resistant obese with hepatic steatosis (IROS), defined as BMI ≥30.0 kg/m^2^ with prediabetes, insulin resistance and IHTG content >5%. We specifically studied people with IROS because hepatic steatosis commonly accompanies insulin resistance in people with obesity (8, 9) and including those without hepatic steatosis would increase the heterogeneity of the cohort. We reasoned that lipidomic profiling of these distinct groups would help distinguish the effects of insulin resistance with hepatic steatosis from the effect of obesity per se on the plasma lipidome and possibly identify novel circulating lipids that could be biomarkers or causes of insulin resistance with steatosis in people with obesity. We hypothesized that lipid classes previously associated with insulin resistance (e.g., triglycerides, diacylglycerols, and ceramides) (2, 10) would be more abundant in the IROS group than the ISO group but would not be different between the ISO and ISL groups, and that plasma eicosanoids would be more abundant in the IROS group than the ISO group.

## Materials and methods

### Participants and study design

A total of 40 people participated in this study. Participants were recruited by using the Volunteers for Health database at Washington University School of Medicine and by local postings. All study procedures were conducted in the Clinical Translational Research Unit (CTRU) at Washington University School of Medicine. Potential participants completed an initial screening evaluation that included a medical examination, standard blood tests, a 2-h oral glucose tolerance test (OGTT), body composition assessments [fat mass and fat-free mass (FFM) by using dual-energy X-ray absorptiometry (Lunar iDXA, GE HealthCare) and intrahepatic triglyceride (IHTG) by using magnetic resonance imaging (3T MAGNETOM Vida, Siemens)] and a hyperinsulinemic-euglycemic clamp procedure (HECP). Participants were included if they met criteria for one of three groups: i) ISL (n = 13): body mass index (BMI) 18.5–24.9 kg/m^2^ and normal fasting plasma glucose (<100 mg/dl), oral glucose tolerance (2-h glucose <140 mg/dl), IHTG content (<5%), plasma triglycerides (<150 mg/dl), and glucose infusion rate (GIR) during the HECP >50 μg per kilogram fat-free mass (FFM) divided by plasma insulin concentration (I) (expressed as μg/kg FFM/min/μU/ml); ISO (n = 14): BMI 30.0–49.9 kg/m^2^ and normal fasting plasma glucose, oral glucose tolerance, IHTG content, plasma triglycerides, and GIR/I > 50 μg/kg FFM/min/μU/ml; and IROS (n = 13): BMI 30.0–49.9 kg/m^2^, impaired fasting glucose or oral glucose tolerance, high IHTG content (≥5%) and GIR/I ≤ 40 μg/kg FFM/min/μU/ml. No participant had a history of diabetes, liver disease other than hepatic steatosis, major chronic cardiac, renal, or pulmonary disease, was pregnant or lactating, consumed alcohol in excess of 14 drinks per week for women or 21 drinks per week for men, or used tobacco products. No participants took medications that could affect plasma lipids. Plasma samples for lipidomic analysis were obtained from some participants as part of their participation in another study (9). All participants provided written informed consent before enrolling in this study, which complied with the principles of the Declaration of Helsinki, was approved by the Washington University Institutional Review Board and was registered in clinicaltrials.gov (NCT04131166 and NCT02706262).

### Insulin sensitivity

Participants were admitted to the CTRU in the early evening two days before conducting the HECP. Participants received 4 standard meals, each containing one-third of their estimated energy requirement (11) at 1900 h on day 1 and 0700 h, 1300 h, and 1900 h on day 2. On day 3, after participants fasted for ∼12 h overnight, catheters were inserted into a radial artery to obtain arterial blood samples and a forearm vein to infuse dextrose and glucose tracer. Whole-body (primarily skeletal muscle) and hepatic insulin sensitivity were measured by using a HECP in conjunction with stable isotopically labeled glucose tracer infusion, as previously described (9). At 0700 h, a primed (8.0 μmol/kg) continuous (0.08 μmol/kg/min) infusion of [U-^13^C]glucose (Cambridge Isotope Laboratories) was started. After 210 min (basal period), glucose infusion was stopped and insulin infused at a rate of 50 mU insulin/m^2^ body surface area/min. Euglycemia (100 mg/dl) was maintained during the HECP by infusing 20% dextrose spiked to 1% with [U-^13^C]-glucose. Whole-body insulin sensitivity was calculated as the rate of glucose disposal per kilogram FFM during the last 20 min (160–180 min of insulin infusion) of the HECP divided by plasma insulin concentration (glucose R_d_/I) (12). Plasma glucose tracer-to-tracee ratio (TTR) was determined by using gas chromatography-mass spectrometry, as previously described (13). The hepatic insulin sensitivity index (HISI) was calculated as the inverse of the product of the plasma insulin concentration and endogenous glucose rate of appearance (R_a_) during the basal period of the HECP, as described (9).

### Plasma glucose and insulin concentrations

Plasma glucose was measured by using an automated glucose analyzer (Yellow Springs Instruments, Yellow Springs, OH). Plasma insulin concentration was measured by using an electrochemiluminescence assay (Elecsys 2010, Roche Diagnostics, Indianapolis, IN).

### Plasma lipidomics

An arterial blood sample for lipidomic analysis was obtained through an indwelling catheter at 0700 h in the morning of the HECP after participants fasted for ∼11.5 h overnight. The blood sample was immediately placed in a chilled EDTA tube, plasma separated by centrifugation, and the plasma stored at −80° C until subsequent analyses. Basic lipid panels were analyzed by using the COBAS 6000 automated chemistry analyzer (Roche Diagnostics) in the Washington University Core Laboratory for Clinical Studies.

For complex lipid analysis, 20 μl of human plasma was spiked with a mix of deuterated internal standards (Equisplash, Avanti Polar Lipids, 330,371). The mix contained PC 15:0/18:1-d_7_, LPC 18:1-d_7_, PE 15:0/18:1-d_7_, LPE 18:1-d_7_, PG 15:0/18:1-d_7_, PI 15:0/18:1-d_7_, PS 15:0/18:1-d_7_, TG 15:0/18:1-d_7_/15:0, DAG 15:0/18:1-d_7_, MG 18:1-d_7_, CE 18:1-d_7_, SM d18:1/18:1-d_9_, and Cer d18:1-d_7_/15:0 at equal concentrations. All lipids were measured according to the method described previously (14). Samples were extracted using a modified butanol-methanol method. A Vanquish UPLC (Thermo Fisher Scientific) was interfaced with a Q-Exactive mass spectrometer (Thermo Fisher Scientific). A Waters T3 1.6 μM 2.1 mm × 150 mm column was used for chromatographic separation with a step gradient from 25% buffer A (10 mM ammonium formate and 0.1% formic acid in water) to 100% buffer B (70/30 isopropanol/acetonitrile with 10 mM ammonium formate and 0.1% formic acid) over 35 min with a flow rate of 0.3 ml/min. Lipid analytes were analyzed using a data dependent acquisition Top N scan of 8 with an NCE of 30 in negative mode and an NCE of 25 in positive mode and a scan range of 200–1200 m/z. MS1 resolution was set at 70,000 (full width at half maximum at m/z 200) with an automatic gain control target of 1e6 and maximum ion injection time of 200 ms. MS2 resolution was set to 17,500 with an automatic gain control target of 5e4, fixed first mass of 80 m/z, and maximum ion injection time of 50 ms. The isolation width was set at 1.2; dynamic exclusion was set at 3 s. Lipid identification and quantification was carried out with Lipidomic Data Analyzer (LDA), software that adheres to the strict guidelines established by the lipidomics community with respect to accurate annotation and quantitation of complex lipids (15, 16). This widely used software (17, 18, 19) is freely available (20) and can be downloaded together with the list of all m/z values of the molecular and product ions. The Reporting Checklist of the Lipidomics Standards Initiative (21) is available at https://doi.org/10.5281/zenodo.18134095 and is provided as Supplemental Information.

For eicosanoid and related PUFA analysis, a mix of 23 deuterated internal standards was spiked into 50 μl of human plasma. The mix contained 6k-PGF_1α_-d_4_, TXB_2_-d_4_, PGF_2α_-d_4_, PGE2-d_4_, PGD2-d_4_, 15d-PGJ2-d_4_, dhk-PGF_2α_-d_4_, dhk-PGD_2_-d_4_, 8-iso-PGF_2a_-d_4_, LTB_4_-d_4_, 5-HETE-d_8_, 12-HETE-d_8_, 15-HETE-d_8_, 20-HETE-d_8_, 9-HODE-d_4_, 13-HODE-d_4_, 8,9-EET-d_11_, 11,12-EET-d_11_, 14,15-EET-d_11_, 9,10-diHOME-d_4_, 12,13-diHOME-d_4_, LTE_4_-d_5_, and arachidonic acid-d_8_ in equal concentrations. A SCIEX Triple Quad mass spectrometer interfaced with a Waters Acquity UPLC was employed as previously described in detail (6, 22). Eicosanoids were extracted by solid phase extraction using Phenomenex Strata-X polymeric reversed phase columns. Samples were brought to dryness and taken up in buffer A (water/acetonitrile/acetic acid 60/40/0.02, v/v/v). A Waters BEH Shield 1.7 μM 2.1 mm × 100 mm column was used for chromatographic separation with a step gradient starting with 100% buffer A to 100% buffer B (acetonitrile/isopropanol 50/50, v/v) over 6 min and a flow rate of 0.5 ml/min. Standard curves were obtained in parallel using identical conditions. Data analysis was performed with Analyst and Multiquant software packages.

Plasma non-esterified fatty acid (NEFA) concentrations were determined as previously described (23). Plasma was spiked with heptadecanoic acid and extracted with heptane. After acetone precipitation of plasma proteins and evaporation of solvent, NEFAs were derivatized with iodomethane to fatty acid methyl esters (FAME). For quantitation, an Agilent 6890N Network GC system was interfaced to an Agilent 5973 Inert MSD with a Turbo EI source. Chromatographic separation was performed on an Omega-Wax column (Supelco; 30 m × 0.25 mm, 0.25 μm film thickness). The GC oven was initially held at 180°C for 0.5 min, then ramped to 200°C at 40 °C/min, held for 20 min, then ramped to 250°C at 70 °C/min, then held for 2 min, resulting in a total runtime of 23.7 min. Helium was used as the carrier gas at a constant flow rate of 1.5 ml/min. The MS source temperature was set to 230°C and the quadrupole temperature to 150°C. Mass spectra were acquired in scan mode over a mass range of 50–500 amu.

### Lipidomics data analysis

Lipid species with MS/MS identification at acyl chain resolution were included in the analysis. For analysis of individual complex lipid species, species not detected in more than 50% of samples (n = 13) were removed. Relative abundances were converted to absolute concentrations (pmol/ml plasma) by using the deuterated class-specific internal standard and assuming a uniform ionization response across species within each lipid class. Missing values (13.8% of values) were replaced by one-fifth of the minimum positive value of that lipid variable. No features were filtered out. The complex lipids analyzed are listed in Supplemental Table 1. Data were scaled for principal component analysis by converting to a Z-distribution. Principal component analysis was performed by using the prcomp function in base R. For volcano plot analyses of individual complex lipid and eicosanoid species, species with *P* < 0.005 were considered significantly different. Only eicosanoid species detected in more than 50% of samples (45 of 84 species) were included in the analysis. When values for specific eicosanoids were not detected, a value that was one-fifth of the minimum positive value was imputed.

### Statistical analysis

Participant characteristics, summed lipid class, and NEFA data were tested for normality by using the Kolmogorov-Smirnov test. Normally distributed variables are reported as mean ± SD, and non-normally distributed variables are reported as median (IQR). Participant characteristics, lipid subclasses, individual complex lipid species, and NEFA data were analyzed by using one-way ANOVA with Tukey’s post hoc test or the Kruskal–Wallis test with Dunn’s post hoc test used to adjust for multiple comparisons and identify significant group differences as appropriate. Associations between lipid subclass or individual lipids and insulin sensitivity, BMI, or IHTG content were determined by using Spearman’s ρ, applying the Benjamini-Hochberg correction for multiple comparisons. PC1 principal component analysis scores were compared by using one-way ANOVA with Tukey’s HSD post-hoc testing. The numbers of individual lipid species correlated with insulin sensitivity or BMI were compared by using McNemar’s test. A previously described (24) three-lipid classifier (PI 32:1 [16:0/16:1], PE 36:1 [18:0/18:1], and Cer d42:0 [d18:0/n24:0]) was evaluated for discrimination of IROS status. A logistic regression model was fitted with these three lipid features as predictors and IROS status (binary outcome) as the dependent variable. Model discrimination was assessed by using receiver operating characteristic (ROC) curve analysis with the area under the curve (AUC) calculated by using the DeLong method. Relative abundances of each eicosanoid species were transformed to Z-distributions and the average eicosanoid Z-score for each participant was calculated. Average eicosanoid Z-scores in the ISL group were compared to the combined ISO and IROS groups by a two-sided independent-samples *t* test. Statistical analyses were performed by using GraphPad Prism version 10 and R version 4.5.

## Results

### Participant characteristics

There were no differences in age or sex between the ISL, ISO and IROS groups, no differences in BMI, percent body fat mass or fat-free mass between the ISO and IROS groups, and no differences in IHTG content, fasting plasma glucose, 2-h OGTT glucose, fasting plasma insulin, total cholesterol, HDL-cholesterol, LDL-cholesterol, fasting plasma triglycerides, or whole-body insulin sensitivity between the ISL and ISO groups (Table 1). Compared to the ISO group, the IROS group had higher IHTG content, fasting plasma glucose, 2-h OGTT glucose, fasting plasma insulin, and fasting plasma triglycerides, lower HDL-cholesterol, and more than 2-fold lower insulin sensitivity.

**Table 1:** Participant characteristics

Variable	ISL	ISO	IROS	*P* value
Age (yr)	35.9 ± 10.0	41.3 ± 7.8	40.1 ± 7.5	0.24
Sex (M/F)	5/8	4/10	4/9	0.91
BMI (kg/m^2^)	22.9 (21.3–24.1)	34.5 (33.5–38.9)^a^	34.7 (32.7–41.7)^a^	**<0.0001**
Fat-free mass (kg)	44.1 ± 6.5	55.1 ± 13.1^a^	56.2 ± 10.3^a^	**0.0085**
Body fat (%)	29.2 (23.8–31.7)	49.5 (41.9–53.3)^a^	45.0 (39.5–50.7)^a^	**<0.0001**
IHTG content (%)	1.9 ± 0.6	2.8 ± 0.7	16.5 ± 7.3^a^^,^^b^	**<0.0001**
Fasting glucose (mg/dl)	85 ± 4	88 ± 5	98 ± 10^a^^,^^b^	**<0.0001**
2-h OGTT glucose (mg/dl)	93 ± 19	98 ± 16	171 ± 29^a^^,^^b^	**<0.0001**
Fasting insulin (μU/ml)	5.0 (2.9–6.0)	7.4 (5.7–11.2)	17.6 (16.3–27.2)^a^^,^^b^	**<0.0001**
Insulin sensitivity: Glucose R_d_/I (nmol/kg FFM/min)/(μU/ml)	593 ± 121	570 ± 173	200 ± 26^a^^,^^b^	**<0.0001**
Hepatic insulin sensitivity index: 1000/((μmol/kg FFM/min)^a^(μU/ml)	11.6 ± 4.0	7.9 ± 4.0	3.1 ± 0.9^a^^,^^b^	**<0.0001**
Total cholesterol (mg/dl)	174 ± 23	174 ± 43	182 ± 22	0.74
HDL cholesterol (mg/dl)	64 ± 15	59 ± 17	42 ± 9^a^^,^^b^	**0.0007**
LDL cholesterol (mg/dl)	95 ± 21	102 ± 34	103 ± 20	0.74
Triglycerides (mg/dl)	66 (49–88)	66 (50–71)	147 (111–270)^a^^,^^b^	**<0.0001**

Bold denotes *P* < 0.05.

ISL, insulin-sensitive and lean; ISO, insulin-sensitive with obesity; IROS, insulin-resistant with obesity and hepatic steatosis; M, male; F, female; BMI, body mass index; IHTG, intrahepatic triglyceride; OGTT, oral glucose tolerance test; FFM, fat-free mass. HDL, high-density lipoprotein. LDL, low-density lipoprotein. Values are mean ± SD or median (IQR). Group sizes are n = 13 for ISL and IROS and n = 14 for ISO. *P* values by one-way ANOVA followed by Tukey’s post hoc test for normally distributed variables, Kruskal-Wallis test followed by Dunn’s post hoc test for non-normally distributed variables, or Fisher’s exact test for categorical variables.

a *P* < 0.05 versus ISL.

b *P* < 0.05 versus ISO.

### Plasma complex lipids

**Fig. 1:**
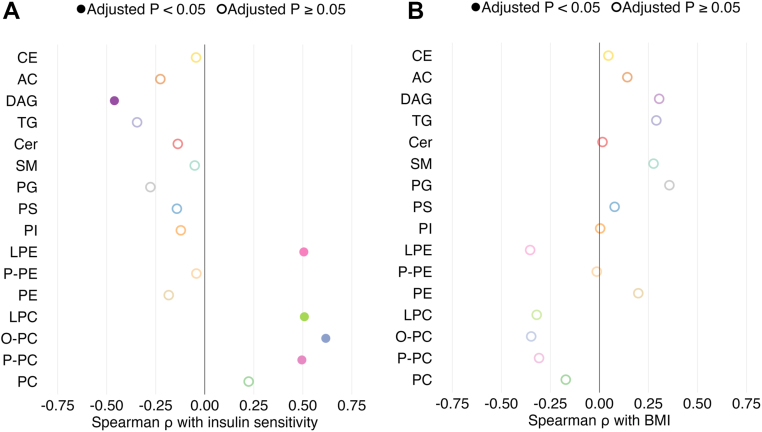
Correlation of complex lipid subclasses with insulin sensitivity and BMI. Spearman ρ coefficients relating lipid classes to insulin sensitivity (A, assessed as glucose R_d_/I) and BMI (B) in the full set of 40 participants in ISL, ISO, and IROS groups. *P* values were adjusted for multiple comparisons by using the Benjamini-Hochberg method.

A total of 772 individual lipid species in 16 subclasses (15 with more than one species) were identified at the level of acyl chain composition (Supplemental Table 1); 759 of these lipids were detected in ≥50% of samples. When expressed as the sum of individual species within subclasses, no lipid subclasses were significantly different between the ISL and ISO groups (Table 2). However, the total abundances of three lipid subclasses [phosphatidylethanolamine (PE), triglyceride (TG), and diacylglycerol (DAG)] were significantly greater in the IROS group than the ISO group (Table 2). In the full cohort (ISL, ISO, and IROS groups), four lipid subclasses were positively correlated with insulin sensitivity: total P-PC, O-PC, LPC, and lysophosphatidylethanolamine (LPE); and one lipid subclass (total DAG) was negatively correlated with insulin sensitivity (Fig. 1A). No lipid classes were significantly correlated with BMI (Fig. 1B).

**Table 2:** Complex lipid subclasses in ISL, ISO, and IROS groups

Lipid Subclass	ISL	ISO	IROS	*P* value
Phosphatidylcholine (PC)	1040 (991–1151)	890 (767–1178)	979 (920–1064)	0.27
Alkenyl ether-containing PC (P-PC)	63 (46–71)	46 (42–60)	37 (34–46)^a^	**0.0086**
Alkyl ether-containing PC (O-PC)	97 ± 20	87 ± 22	69 ± 16^a^	**0.0040**
Lysophosphatidylcholine (LPC)	181 ± 36	170 ± 43	146 ± 30	0.059
Phosphatidylethanolamine (PE)	24 ± 9	21 ± 9	40 ± 24^a^^,^^b^	**0.0083**
Alkenyl ether-containing PE (P-PE)	29 ± 13	26 ± 8	26 ± 7	0.59
Lysophosphatidylethanolamine (LPE)	9.0 ± 1.9	7.7 ± 2.1	6.8 ± 1.6^a^	**0.018**
Phosphatidylinositol (PI)	34 (34–39)	33 (30–39)	43 (21–47)	0.81
Phosphatidylglycerol (PG)	0.030 ± 0.025	0.044 ± 0.019	0.078 ± 0.055^a^	**0.0053**
Phosphatidylserine (PS)	0.18 (0.12–0.27)	0.29 (0.08–0.57)	0.27 (0.15–2.27)	0.43
Sphingomyelin (SM)	433 ± 55	474 ± 90	470 ± 75	0.32
Ceramide (Cer)	6.5 ± 0.9	6.0 ± 1.9	7.8 ± 2.4	0.057
Triglyceride (TG)	526 (332–669)	621 (448–770)	1245 (779–1857)^a^^,^^b^	**0.0041**
Diacylglycerol (DAG)	58 ± 27	57 ± 17	134 ± 66^a^^,^^b^	**<0.0001**
Acylcarnitine (AC)	0.42 ± 0.09	0.43 ± 0.08	0.42 ± 0.07	0.93
Cholesteryl ester (CE)	3011 ± 356	3269 ± 1081	3154 ± 594	0.67

Bold denotes *P* < 0.05.

Lipid subclass totals are expressed as sum of species; all units are μmol/L. Values are means ± SD or median (IQR). Group sizes are n = 13 for ISL and IROS and n = 14 for ISO. *P* values by one-way ANOVA followed by Tukey’s post hoc test for normally distributed variables, Kruskal-Wallis test followed by Dunn’s post hoc test for non-normally distributed variables.

a *P* < 0.05 versus ISL.

b *P* < 0.05 versus ISO.

**Fig. 2:**
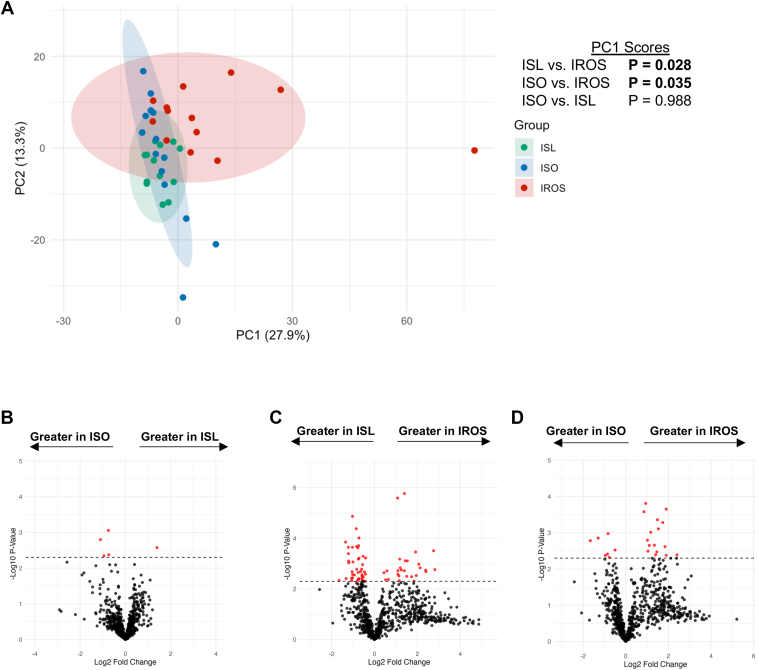
Comparison of individual complex lipid species in ISL, ISO, and IROS groups. Complex lipid species were analyzed after removing lipids with missing values in >50% of samples and imputing missing data. A: Principal component analysis of the ISL, ISO, and IROS groups. PC1 scores were compared by one-way ANOVA (omnibus *P* = 0.016) with Tukey HSD post-hoc test results shown at right. B–D: Volcano plots comparing complex lipid abundances in ISL and ISO groups (B), ISL and IROS groups (C) or ISO and IROS groups (D) using a significance threshold of *P* < 0.005 (dashed lines).

Principal component analysis (PCA) of individual complex lipid species (n = 759 features) found the IROS group was significantly different from the ISO and ISL groups in PC1 space, but that the ISO and ISL groups were not different from each other (Fig. 2A). Volcano plots showed that the abundances of only four complex lipid species (one ceramide and three sphingomyelin species) were greater in the ISO than the ISL group and only one lipid species (a plasmalogen-PE) was greater in the ISL than the ISO group (Fig. 2B, Table 3). In contrast, the abundances of forty-two lipid species (thirty-nine phospholipids, two sphingolipids, and one cholesteryl ester species) were greater in the ISL than the IROS group and twenty-seven lipid species (eleven DAGs, nine sphingolipids, six phospholipids, and one cholesteryl ester) were greater in the IROS than the ISL group (Fig. 2C, Table 3). Sixteen lipid species (ten DAGs, five sphingolipids, and one TG species) were greater in the IROS group than the ISO group (Fig. 2D, Table 3). Seven lipids (all phospholipids) were significantly more abundant in the ISO group than the IROS group (Fig. 2D, Table 3).

**Table 3:** Differentially abundant complex lipids in ISL, ISO, and IROS groups

ISO > ISL	ISL > ISO	ISL > IROS	IROS > ISL	ISO > IROS	IROS > ISO
Cer t42:1 (d18:1/h24:0)	P-PE 42:3 (24:1/18:2)	PC 37:5 (17:0_20:5)	Cer t42:2 (d18:1/h24:1)	PC 37:5 (17:0_20:5)	DG 34:3 (16:0_18:3)
SM d43:3 (d20:1/n23:2)		PC 35:3 (17:1_18:2)	Cer d36:0 (n18:0_d18:0)	LPC 19:1	DG 38:5 (18:2_20:3)
SM d40:4 (d14:3_n26:1)		PI 39:4 (19:0_20:4)	Cer t41:1 (d18:1/h23:0)	PI 38:5 (18:1/20:4)	TG 52:7 (16:2_18:2_18:3)
SM d40:3 (d16:1/n24:2)		PC 39:5 (17:0_22:5)	PE 40:4 (18:0_22:4)	O-PC 40:7 (18:1/22:6)	DG 36:1 (18:0_18:1)
		O-PC 32:1 (16:0/16:1)	DG 36:1 (18:0_18:1)	LPC 19:0	DG 36:5 (18:2_18:3)
		PC 37:2 (18:2_19:0)	DG 35:2 (17:1_18:1)	P-PC 34:1 (16:0/18:1)	DG 34:2 (16:0_18:2)
		LPC 19:1	DG 40:7 (18:1_22:6)	P-PC 32:0 (16:0/16:0)	DG 36:4 (18:2_18:2)
		O-PC 36:2 (18:0/18:2)	DG 38:5 (18:2_20:3)		Cer d36:0 (n18:0_d18:0)
		O-PC 40:5 (20:1/20:4)	DG 34:1 (16:0_18:1)		DG 38:4 (18:1_20:3)
		O-PC 40:7 (18:1/22:6)	PG 36:2 (18:0/18:2)		DG 36:2 (18:1_18:1)
		LPC 19:0	DG 34:2 (16:0_18:2)		DG 36:3 (18:1_18:2)
		O-PC 42:2 (24:0/18:2)	DG 38:4 (18:1_20:3)		DG 38:5 (18:1_20:4)
		PC 37:1 (18:1_19:0)	SM d36:0 (d18:0/n18:0)		Cer d42:0 (d18:0/n24:0)
		P-PC 34:1 (16:0/18:1)	SM d40:0 (d18:0/n22:0)		Cer d40:0 (d18:0/n22:0)
		CE 22:4	DG 36:2 (18:1_18:1)		SM d40:0 (d18.0/n22:0)
		P-PC 36:3 (18:1/18:2)	Cer t42:1 (d18:1/h24:0)		SM d42:0 (d18.0/n24:0)
		O-PC 36:2 (18:1/18:1)	Cer d42:0 (d18:0/n24:0)		
		P-PC 34:2 (16:0/18:2)	Cer d40:0 (d18:0/n22:0)		
		O-PC 36:1 (18:0/18:1)	PI 32:1 (16:0/16:1)		
		LPC 24:1	DG 36:4 (18:2_18:2)		
		LPC 18:2	DG 38:5 (18:1_20:4)		
		PC 37:2 (19:0/18:2)	DG 36:3 (18:1_18:2)		
		SM t40:1 (t14:1_n26:0)	SM d42:0 (d18:0/n24:0)		
		PC 31:0 (15:0_16:0)	PI 40:5 (18:0/22:5)		
		O-PC 34:1 (16:0/18:1)	CE 20:3		
		O-PC 36:3 (18:1/18:2)	PC 40:5 (18:0/22:5)		
		LPC 20:1	PC 38:3 (18:0/20:3)		
		LPC 18:1			
		PC 33:0 (16:0_17:0)			
		PC 33:2 (15:0/18:2)			
		PC 39:4 (19:0_20:4)			
		LPC 20:2			
		PC 33:0 (16:0_17:0)			
		Cer t41:0 (t18:0/n23:0)			
		O-PC 40:4 (20:0/20:4)			
		O-PC 34:1 (18:1/16:0)			
		P-PC 32:0 (16:0/16:0)			
		LPC 20:4			
		LPE 20:4			
		O-PC 38:5 (18:1/20:4)			
		PI 36:1 (18:0/18:1)			
		PC 35:2 (17:0/18:2)			

Complex lipids with differential abundance for each pairwise comparison using a significance threshold of *P* < 0.005. Lipids are shown in order of greatest to least fold difference between groups.

**Fig. 3:**
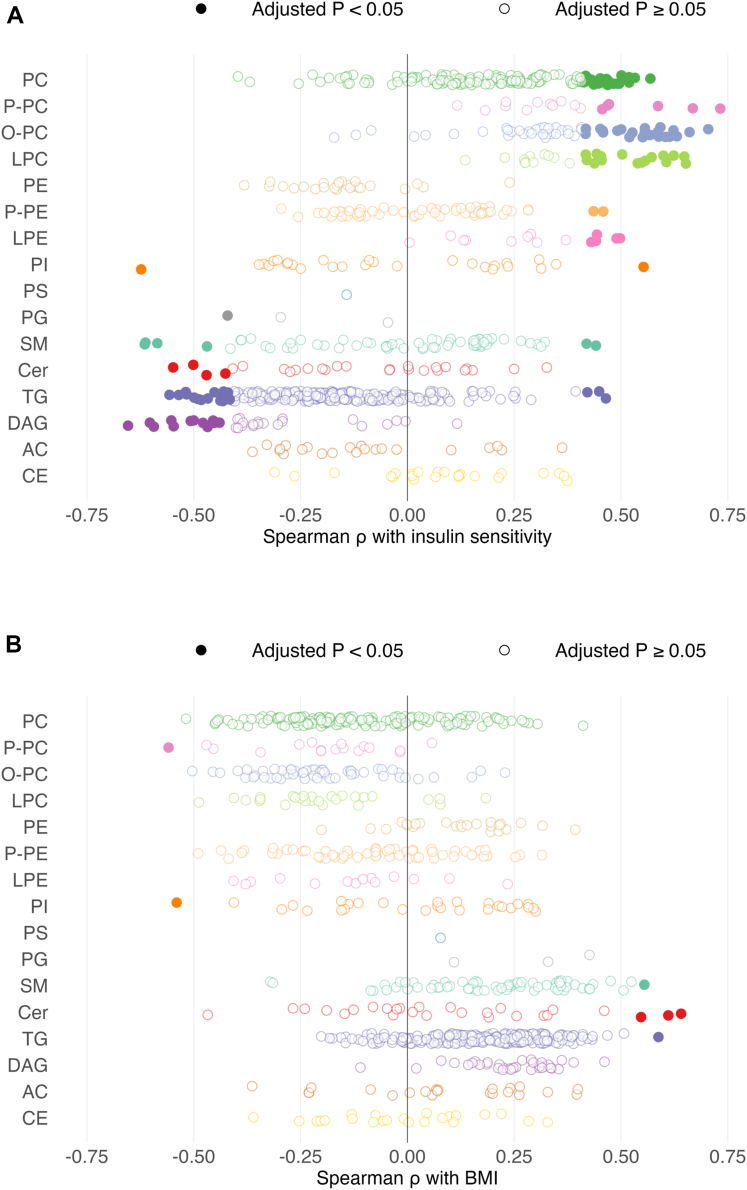
Correlation of individual lipids with insulin sensitivity or BMI. Spearman ρ correlation coefficients relating individual lipid abundance to insulin sensitivity (panel A, assessed as glucose R_d_/I) or BMI (panel B). Each circle represents a distinct complex lipid species within a lipid subclass, shown at left. Significant (BH-adjusted *P* < 0.05) correlations are shown in filled circles; non-significant (BH-adjusted *P* ≥ 0.05) correlations are shown in unfilled semitransparent circles. N = 759 complex lipids, 40 participants.

**Fig. 4:**
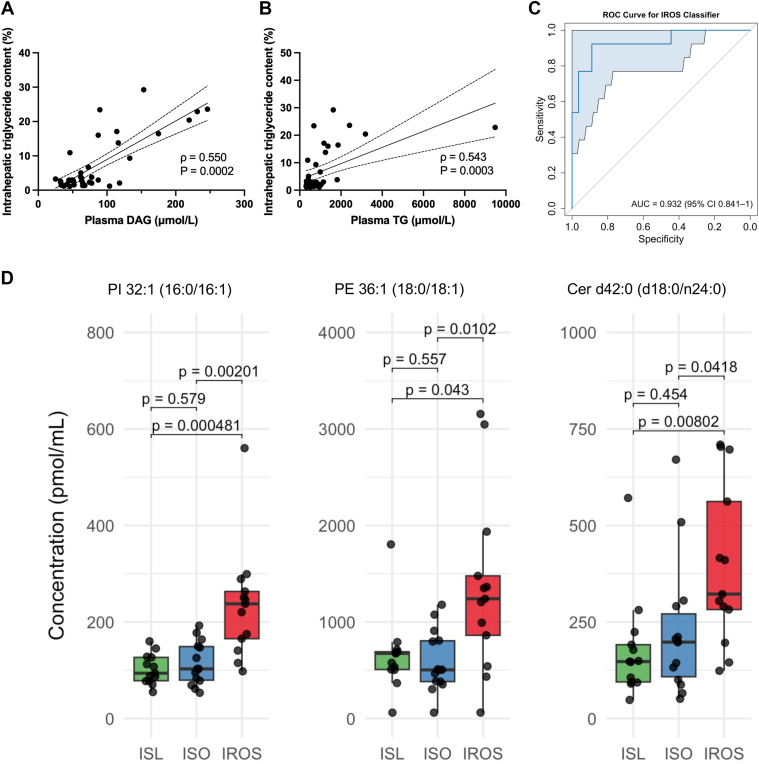
Association of plasma complex lipids with intrahepatic triglyceride. A–B: Relationship between plasma DAG (A) and TG (B), expressed as sum of species, and intrahepatic triglyceride content. Linear trendline with 95% confidence band is plotted. C: Receiver–operating characteristic (ROC) curve with 95% CI for discriminating IROS from ISO and ISL by using the three lipid species (shown in Figure 4D) previously found to identify hepatic steatosis in children (24). (D) Concentrations of the three lipid species comprising the ROC panel in ISL, ISO, and IROS groups. Boxplots show median and interquartile range (IQR). Whiskers extend to the most extreme values within 1.5 x IQR. *P* values by Kruskal-Wallis test with Dunn’s post hoc test. N = 13 ISL, 14 ISO, 13 IROS.

Of 759 individual complex lipid species that were assessed, more lipids (n = 124) were significantly correlated with insulin sensitivity than with BMI (n = 7) after adjustment for multiple comparisons (*P* < 3.2 x 10^-25^, McNemar’s test) (Fig. 3). The lipid species that was most strongly correlated with insulin sensitivity was P-PC 34:1 (16:0/18:1) (ρ = 0.73, adjusted *P* = 0.0003) (Fig. 3B). Several lipid subclasses had a large proportion (>33%) of species that were positively associated with insulin sensitivity after adjustment for multiple comparisons, specifically LPC (16/27 = 59.3% of species), O-PC (23/54 = 42.6% of species), LPE (5/14 = 35.7% of species), and P-PC (6/17 = 35.3% of species). Conversely, the only lipid subclass that had a large proportion (>25%) of species that were negatively associated with insulin sensitivity was DAG (14/34 = 41.2% of species). A greater proportion of plasma DAG species (23/34 = 67.6%) than plasma TG species (72/225 = 32.0%) were associated with intrahepatic triglyceride (IHTG) content (*P* = 8.5 x 10^-7^, McNemar’s test), and total plasma DAGs and TGs (sum of species) were positively associated with IHTG content (Fig. 4A–B). The plasma DAG species most strongly correlated with IHTG content were DAG 36:1 (18:0_18:1) (ρ = 0.65, adjusted *P* = 0.001) and DAG 34:1 (16:0_18:1) (ρ = 0.61, adjusted *P* = 0.002). The plasma TG species most strongly correlated with IHTG content were TG 58:8 (18:1_18:2_22:5) (ρ = 0.64, adjusted *P* = 0.001) and TG 54:2 (18:0_18:1_18:1) (ρ = 0.63, adjusted *P* = 0.002). Three lipid species, namely Cer d42:0 (d18:0/n24:0), PE 36:1 (18:0/18:1), and PI 32:1 (16:0/16:1), that predicted hepatic steatosis in children and adolescents (24) were greater in the IROS group than the ISO and ISL groups and also successfully predicted the IROS group in our adult participants as demonstrated by an area under the receiver operating characteristic curve of 0.932 (95% CI, 0.841–1) (Fig. 4C–D).

### Plasma eicosanoids

**Fig. 5:**
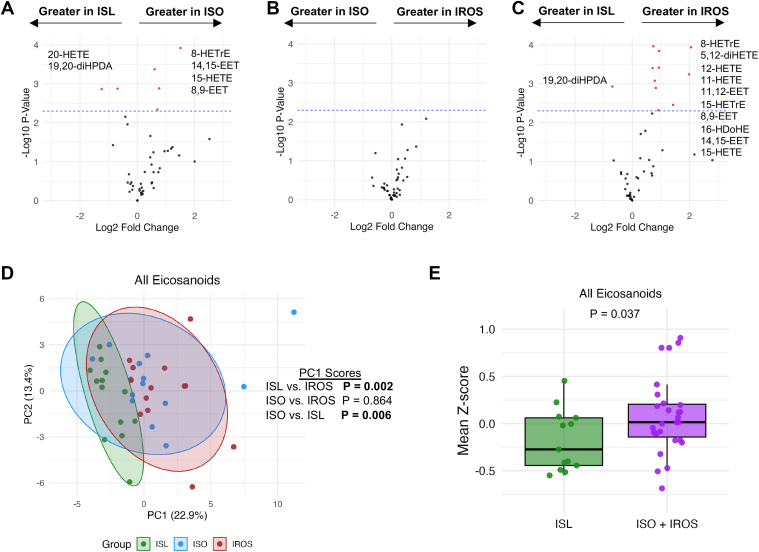
Plasma eicosanoids in ISL, ISO, and IROS groups. Relative abundances of individual eicosanoid species were analyzed after removing eicosanoids with missing values in >50% of samples and imputing missing data. (A–C) Volcano plots comparing lipid abundances in (A) ISL and ISO groups, (B) ISO, and IROS groups, and (C) ISL and IROS groups using a significance threshold (dashed line) of *P* < 0.005, with identities of significantly different eicosanoids shown in order of greatest fold difference between groups. D: Principal component analysis of the ISL, ISO, and IROS groups for all eicosanoids. PC1 scores were compared by one-way ANOVA with Tukey HSD post-hoc testing; omnibus ANOVA *P* value was <0.01. E: Relative abundances of each eicosanoid species were transformed to Z-distributions, and the mean eicosanoid Z-score for each participant is shown, comparing the ISL group to the combined ISO and IROS groups.

Eighty-four of the 136 plasma eicosanoids that were measured were present in at least one sample. We analyzed the 45 eicosanoids that were detected in >50% of plasma samples. A volcano plot analysis of pairwise comparisons of the ISL, ISO, and IROS groups showed there were no significant differences in the abundance of any eicosanoids between the ISO and IROS groups, but the abundance of 6 of 45 eicosanoids were different between the ISL and ISO groups and the abundance of 11 of 45 eicosanoids were different between the ISL and IROS groups (Fig. 5A–C). Consistent with these results, a principal component analysis found the ISL group clustered differently in PC1 space than the ISO or IROS groups (Fig. 5D). The normalized mean eicosanoid abundance was greater in participants with obesity (ISO and IROS groups combined) than in the ISL group (Fig. 5E).

### Plasma non-esterified fatty acids

Total plasma NEFA concentration, comprised of the sum of eight species, tended to be lower in the ISO group than the IROS group, but the difference was not statistically significant (Table 4). All of the most abundant plasma NEFA species (palmitate 16:0, oleate 18:1, linoleate 18:2, and stearate 18:0) also tended to be lower in the ISO than the IROS group, but none of these differences were statistically significant.

**Table 4:** Plasma nonesterified fatty acid profiles

NEFA (μmol/L)	ISL	ISO	IROS	ANOVA *P* value
14:0	6.2 ± 1.6	6.8 ± 1.9	6.2 ± 1.7	0.64
16:0	122.6 ± 37.8	102.6 ± 32.0	130.1 ± 24.6	0.08
16:1	14.8 ± 8.5	15.1 ± 9.3	14.0 ± 5.0	0.93
18:0	44.8 ± 13.1	35.0 ± 10.4	42.3 ± 9.9	0.07
18:1	193.8 ± 64.9	146.6 ± 51.2	175.9 ± 38.5	0.07
18:2	78.7 ± 30.4	61.0 ± 23.7	77.0 ± 15.9	0.12
18:3	7.2 ± 2.9	5.9 ± 1.7	6.2 ± 1.6	0.27
20:4	4.0 ± 1.1	3.7 ± 1.6	3.7 ± 1.4	0.83
Sum of Species	472.1 ± 150.4	376.7 ± 120.1	455.4 ± 87.5	0.11

NEFA, non-esterified fatty acids; ISL, insulin-sensitive and lean; ISO, insulin-sensitive with obesity; IROS, insulin-resistant with obesity and hepatic steatosis; Values are mean ± SD. Group sizes are n = 13 ISL, n = 14 ISO, n = 13 IROS.

## Discussion

Plasma lipids represent important biomarkers and potential mechanisms linking obesity with insulin resistance and cardiometabolic diseases. In the present study we evaluated the influence of obesity and obesity plus insulin resistance with hepatic steatosis on the plasma lipidome by studying people who were ISL, ISO and IROS. We found a major effect of insulin resistance with hepatic steatosis, but not obesity per se, on the plasma complex lipidome. No complex lipid subclasses were differentially abundant in the ISO and ISL groups, but 19% of subclasses were differentially abundant in the IROS group compared with the ISO group. In contrast, 13% of plasma eicosanoid concentrations were different in the ISO than in the ISL group and none were different in the ISO than in the IROS group. In addition, approximately 16% of plasma complex lipid species were significantly correlated with insulin sensitivity, whereas only 1% were significantly correlated with BMI. These results underscore the independent and complex interactions among adiposity and insulin action in the plasma lipidome and demonstrate that insulin resistance, rather than obesity, affects plasma complex lipids, whereas obesity rather than insulin resistance affects plasma eicosanoids.

The link between obesity and alterations in plasma lipids is complex because of the potential interactive effects of excess adiposity, insulin resistance and hepatic steatosis. Several previous studies have evaluated the plasma lipidome in people who were lean, overweight or obese (10, 25), people who were considered to be insulin sensitive or insulin resistant (2, 10, 26), and people with and without metabolic dysfunction associated steatotic liver disease (MASLD) (6, 27, 28, 29, 30). These studies found that insulin resistance and obesity were associated with increased plasma diacylglycerol and ceramide concentrations and decreased plasma lysophosphatidylcholines and that certain plasma eicosanoids were increased in people with MASLD. Consistent with these results, we found that a large number of plasma complex lipids (124 of 759 of the individual lipid species) correlated with insulin sensitivity, assessed by using the HECP, which is primarily a measure of insulin action in skeletal muscle (12). In addition, total abundance of five out of the 15 lipid subclasses that contained more than one species was significantly associated with insulin action; four subclasses (total P-PC, O-PC, LPC, and LPE) were positively associated with insulin sensitivity, and one subclass (total DAG) was negatively associated with insulin sensitivity. These results are consistent with the findings of a previous study (2) that found total TGs, ceramides, DAGs, and LPCs were associated with insulin sensitivity in people with overweight/obesity after adjusting for age, sex, ethnicity and BMI. Our study extends these findings by also demonstrating that P-PC, O-PC and LPE concentrations are positively associated with insulin sensitivity.

The abundances of several plasma DAGs were greater in the IROS than the ISO group, including 29% of the 34 distinct DAG species and representing 62.5% of total lipid species that were significantly higher in the IROS than the ISO group. In addition, we found plasma DAGs were directly correlated with IHTG content. Our results are consistent with and extend the results from previous studies showing plasma DAG concentrations are increased in people with MASLD (30). Plasma DAG concentrations are coupled (temporal correlation r > 0.66 between identical lipid molecules or lipids with identical backbones in the liver and plasma) to liver DAGs in the postabsorptive state (31) and primarily circulate in triglyceride-rich lipoproteins that have been secreted by the liver (32). An increase in intrahepatic DAGs can cause hepatic insulin resistance by activating protein kinase C-ε, which inhibits insulin receptor kinase activity (33, 34, 35). Our data suggest the increase in plasma DAGs in our IROS groups was caused by increased intrahepatic TG and therefore DAG production, which contributed to hepatic insulin resistance in this group. We also confirmed that a panel of three complex lipids [Cer d42:0 (d18:0/n24:0), PE 36:1 (18:0/18:1), and PI 32:1 (16:0/16:1)] accurately predicted the presence or absence of hepatic steatosis, validating and extending the findings of a study conducted in children and adolescents (24). However, the transition from simple hepatic steatosis to metabolic dysfunction-associated steatohepatitis (MASH) with fibrosis can attenuate the association between plasma DAG concentrations and insulin resistance (36).

About half of the lysophosphatidylcholine (LPC) and ether lipid phosphatidylcholine (P-PC and O-PC) species were associated with increased whole-body insulin sensitivity, in agreement with the results from previous studies (10, 37, 38, 39). Plasma LPC are primarily located in high-density lipoproteins (HDL) (31, 40). HDL size is associated with insulin sensitivity (41) and larger HDLs have proportionally greater phospholipid content (42), which could explain the relationships we observed between certain plasma phospholipids and insulin sensitivity. The plasmalogen ether lipids (P-PC and P-PE) contain a vinyl-ether linkage that is susceptible to oxidative stress; this endogenous antioxidant function has been proposed to protect against insulin resistance, which could explain the negative relationship between plasmalogen ether lipids with diabetes and cardiovascular disease (43, 44). The alkyl ether lipids (O-PC) lack this vinyl ether linkage but can be converted to the plasmalogen P-PC (45).

Obesity per se did not have an important effect on the plasma lipidome. The abundance of only 5 of 759 individual complex lipid species were different between the ISO and ISL groups, which were matched on whole-body insulin sensitivity but were markedly different in adiposity. Our results differ from two previous studies that demonstrated positive correlations between plasma complex lipids and BMI (25, 46). One study found 508 of 706 measured lipid species were significantly associated with BMI after adjustment for age and sex (25) and the other study found 200 of 312 lipid species were associated with BMI after adjustment for 2-h oral glucose tolerance test plasma glucose concentration (46). Our results suggest that the associations between plasma lipids and BMI in previous studies were likely due to the relationship between BMI and insulin sensitivity (47, 48) rather than increased adiposity.

Eicosanoids are important signaling molecules comprised of subfamilies that include prostaglandins, thromboxanes, leukotrienes, lipoxins, and resolvins. Eicosanoids usually act locally and can exert functions that are pro-inflammatory (e.g., platelet aggregation or leukocyte recruitment) or anti-inflammatory (e.g., promoting efferocytosis or modulating cytokine release) (5). Approximately 25% of measured eicosanoids in our participants were greater in the ISO and IROS groups than in the ISL group, but there were no differences in eicosanoid abundance between the ISO and IROS groups. Moreover, eicosanoids in the IRO and ISO groups were distinct from the ISL group by PCA and volcano plot analyses. The eicosanoid that displayed the greatest increase in both ISO and IROS groups compared with the ISL group was 8-HETrE, an ω-6 oxylipin, which is consistent with previous studies that found plasma 8-HETrE was greater in people with obesity (49) and people with obesity and hepatic steatosis (50) than in people who were lean and healthy. Our results suggest that the increase in plasma eicosanoids observed in people with obesity in previous studies was related to increased adiposity rather than insulin resistance and that eicosanoids are unlikely to be the communicating signal linking excess adiposity with insulin resistance in other tissues.

Our study has several limitations. First, only a limited number of isotope-labeled reference standards are available for mass spectrometry-based quantitative analyses of lipids. Our study used the Equisplash cocktail, which contains one isotope-labeled standard per lipid class and category (see Materials and Methods). We assumed that the extraction and ionization efficiency of the individual standards for each lipid class were representative for all lipids in that class (3, 51, 52). Nonetheless, the comparisons between groups are still valid because the same method was used in a single batch run for all samples, accounting for any differences in extraction and ionization efficiency. Second, our study is unable to determine whether the plasma lipidome is affected by sex because of the small number of participants in each study group. Sex-specific differences in the plasma lipidome have been reported previously, showing that more than 80% of lipid subclasses have differential abundance in males and females (25). Third, our study could have missed small differences in complex lipid classes and individual lipid species among groups because of the small size of each group. Fourth, plasma lipids were measured in the postabsorptive state, so our conclusions cannot be generalized to postprandial or insulin-stimulated conditions. Fifth, only one plasma sample was obtained from each subject, so we cannot determine intra-individual, day-to-day variability in the plasma lipidome. Finally, the plasma concentrations of several complex lipids, including medium-long chain (C12-C15) cholesteryl esters, diacylglycerols, and phosphatidylcholines, can be affected by dietary intake (53), which was not controlled in our study.

Our evaluation of the plasma complex lipidome and eicosanoids in three distinct groups of people characterized by adiposity and metabolic health demonstrate that: *i)* insulin resistance and hepatic steatosis, rather than obesity, are associated with alterations in the plasma complex lipidome; *ii)* obesity, rather than insulin resistance/steatosis, is associated with alterations in plasma eicosanoids; and *iii)* increased plasma DAGs correlate directly with IHTG content, supporting the potential use of plasma DAGs as a biomarker of hepatic steatosis. These results underscore the potential for the plasma lipidome to transcend the standard lipid panel in understanding the pathogenesis and pathophysiology of obesity-related metabolic dysfunction.

## Data availability

The data from this manuscript are available upon request from the corresponding author, Samuel Klein (sklein@wustl.edu).

## Supplemental data

This article contains supplemental data.

## Conflict of interest

The authors declare the following financial interests/personal relationships which may be considered as potential competing interests:

S. K. serves on Scientific Advisory Boards for Merck, Abbvie, Verdiva Bio and 89 Bio. E. A. D. is a co-founder and holds equity in LipoNexus, Inc which has licensed technology from the University of California, San Diego. The other authors have nothing to disclose.
