# Supplementary material for: Influence of obesity and insulin resistance with hepatic steatosis on the human plasma lipidome
Source: J Lipid Res. 2025 Dec 26;67(1):100969. doi: 10.1016/j.jlr.2025.100969 (PMC12856300; doi:10.1016/j.jlr.2025.100969)
Supplement: Supplementary Material 2 [file mmc2.pdf]

Contents of Report

Created by https://lipidomicstandards.org, version v2.5.0

Direct Infusion Workflow 1
Overall study design . . . 1
Lipid extraction . . . 1
Analytical platform . . . 2
Quality control . . . 2
Method qualification and validation . . . 2
Reporting . . . 2

Sample Descriptions 2
Influence of obesity and insulin resistance with hepatic steatosis on the human plasma lipidome / Human / Plasma . . . 2

Lipid Class Descriptions 3
1) CAR / Lipid identification . . . 3
1) CAR / Lipid quantification . . . 3
2) TG[M+NH4]+ / Lipid identification . . . 3
2) TG[M+NH4]+ / Lipid quantification . . . 4
3) DG[M+NH4]+ / Lipid identification . . . 4
3) DG[M+NH4]+ / Lipid quantification . . . 4
4) SE[M+NH4]+ / Lipid identification . . . 4
4) SE[M+NH4]+ / Lipid quantification . . . 5
5) Cer[M+HCOO]- / Lipid identification . . . 5
5) Cer[M+HCOO]- / Lipid quantification . . . 5
6) SM[M+HCOO]- / Lipid identification . . . 6
6) SM[M+HCOO]- / Lipid quantification . . . 6
7) PC[M+HCOO]- / Lipid identification . . . 6
7) PC[M+HCOO]- / Lipid quantification . . . 7
8) PC O[M+HCOO]- / Lipid identification . . . 7
8) PC O[M+HCOO]- / Lipid quantification . . . 7
9) PC P[M+HCOO]- / Lipid identification . . . 8
9) PC P[M+HCOO]- / Lipid quantification . . . 8
10) PE[M-H]- / Lipid identification . . . 8
10) PE[M-H]- / Lipid quantification . . . 9
11) PE P[M-H]- / Lipid identification . . . 9
11) PE P[M-H]- / Lipid quantification . . . 9
12) PG[M-H]- / Lipid identification . . . 9
12) PG[M-H]- / Lipid quantification . . . 10
13) PI[M-H]- / Lipid identification . . . 10
13) PI[M-H]- / Lipid quantification . . . 10
14) PS[M-H]- / Lipid identification . . . 11
14) PS[M-H]- / Lipid quantification . . . 11
15) LPC[M+HCOO]- / Lipid identification . . . 11
15) LPC[M+HCOO]- / Lipid quantification . . . 12
16) LPE[M-H]- / Lipid identification . . . 12
16) LPE[M-H]- / Lipid quantification . . . 12
17) LPS[M-H]- / Lipid identification . . . 13
17) LPS[M-H]- / Lipid quantification . . . 13

Direct Infusion Workflow

Overall study design

Title of the study	Influence of obesity and insulin resistance with hepatic steatosis on the human plasma lipidome		
Document creation date	12/16/2025	Principal investigator	Samuel Klein, MD
Institution	Washington University School of Medicine	Corresponding Email	sklein@wustl.edu
Is the workflow targeted or untargeted?	Untargeted	Clinical	Yes

Lipid extraction

Extraction method	2-phase system	pH adjustment	Acetic Acid 1%

2-phase system	BUME	Special conditions	Sonication
Were internal standards added prior extraction?	Yes		

Analytical platform

Ionizaton additives	Ammonium formate, Formic acid	Detector	Mass spectrometer
MS type	Orbitrap	MS vendor	Thermo
Direct type	Syringe	MS Level	MS^2
Mass window for precursor ion isolation (in Da total isolation window)	1.2	Mass resolution for detected ion at MS^2	Low resolution
Resolution at MS^2	Low	Recording mode of raw data at MS^2	Centroid mode
Was/Were additional dimension/techniques used	No		

Quality control

Blanks	No	Quality control	Yes
Type of QC sample	Commercial sample		

Method qualification and validation

Method validation	Yes	Lipid recovery	Yes
Dynamic quantification range	No	Limit of quantitation (LOQ)/Limit of detection (LOD)	Yes
Precision	Yes	Accuracy	Yes
Guidelines followed	None		

Reporting

Are reported raw data uploaded into repository?	Available on request	Are metadata available?	Available on request
Raw data upload	Available on request	Additional comments	The data are available upon request from the corresponding author, Samuel Klein (sklein@wustl.edu).

Sample Descriptions

Influence of obesity and insulin resistance with hepatic steatosis on the human plasma lipidome / Human / Plasma

Storage and collection conditions	Available	Provided preanalytical information	Time to separate plasma/serum (min)
Time to separate plasma/serum (min)	10	Temperature handling original sample	4-8 °C
Instant sample preparation	No	Storage temperature	-80 °C
Additives	EDTA		

Lipid Class Descriptions

1) CAR / Lipid identification

Lipid class	CAR	MS Level for identification	MS^2
Fragments for identification			

Fragment name
Carnitine-C4O2H5

Identification level	sn Position	Isotope correction at MS^2	Type 2
MS^2 verified by standard	Yes	Background check at MS^2	No
Did you presume assumptions for identification?	No	Check on:	Isomeric overlap, Isobaric overlap
Limit of detection	No	Lipid Identification Software	Lipid Data Analyzer (LDA)
Data manipulation	Smoothing	Nomenclature for intact lipid molecule	No
Nomenclature for fragment ions	N/A		

1) CAR / Lipid quantification

Quantitative	Yes	MS Level for quantification	MS^1
Internal lipid standard(s) MS^1			

Internal standard	Endogenous subclass
CAR 18:0-d3	CAR

Type of quantification	Internal standard amount	Response correction	No
Type I isotope correction	Yes	Limit of quantification	Signal threshold
Normalization to reference	Yes	Lipid Quantification Software	Lipid Data Analyzer (LDA)
Batch correction	No		

2) TG[M+NH4]+ / Lipid identification

Lipid class	TG	MS Level for identification	MS^2
Identification level	Molecular species level	MS^2 adduct	[M+NH4]+
Fragments for identification			

Fragment name
-FA1(+HO)-(NH3)
-FA2(+HO)-(NH3)
-FA3(+HO)-(NH3)

Isotope correction at MS^2	Type 2	MS^2 verified by standard	Yes
Background check at MS^2	No	Did you presume assumptions for identification?	No
Check on:	Isomeric overlap, Isobaric overlap	Limit of detection	Signal threshold
Lipid Identification Software	Lipid Data Analyzer (LDA)	Data manipulation	Smoothing
Nomenclature for intact lipid molecule	Yes	Nomenclature for fragment ions	Yes

2) TG[M+NH4]+ / Lipid quantification

Quantitative	Yes	MS Level for quantification	MS^1

Internal lipid standard(s) MS^1

Internal standard	Endogenous subclass
TG 15:0/18:1-d7/15:0	TG

Type of quantification	Internal standard amount	Response correction	No
Type I isotope correction	Yes	Limit of quantification	Signal threshold
Normalization to reference	Yes	Lipid Quantification Software	Lipid Data Analyzer (LDA)
Batch correction	No		

3) DG[M+NH4]+ / Lipid identification

Lipid class	DG	MS Level for identification	MS^2
Identification level	Molecular species level	MS^2 adduct	[M+NH4]+

Fragments for identification

Fragment name
-FA1(-H)-(H2O+NH3)
-FA2(-H)-(H2O+NH3)

Isotope correction at MS^2	Type 2	MS^2 verified by standard	Yes
Background check at MS^2	No	Did you presume assumptions for identification?	No
Check on:	Isomeric overlap, Isobaric overlap	Limit of detection	Signal threshold
Lipid Identification Software	Lipid Data Analyzer (LDA)	Data manipulation	Smoothing
Nomenclature for intact lipid molecule	Yes	Nomenclature for fragment ions	Yes

3) DG[M+NH4]+ / Lipid quantification

Quantitative	Yes	MS Level for quantification	MS^1

Internal lipid standard(s) MS^1

Internal standard	Endogenous subclass
DG 15:0/18:1-d7	DG

Type of quantification	Internal standard amount	Response correction	No
Type I isotope correction	Yes	Limit of quantification	Signal threshold
Normalization to reference	Yes	Lipid Quantification Software	Lipid Data Analyzer (LDA)
Batch correction	No		

4) SE[M+NH4]+ / Lipid identification

Lipid class	SE	MS Level for identification	MS^2
Identification level	Species level	MS^2 adduct	[M+NH4]+

Fragments for identification

Fragment name
Choleseterol-C27H45

Isotope correction at MS^2	Type 2	MS^2 verified by standard	Yes
Background check at MS^2	No	Did you presume assumptions for identification?	No
Check on:	Isomeric overlap, Isobaric overlap	Limit of detection	Signal threshold
Lipid Identification Software	Lipid Data Analyzer (LDA)	Data manipulation	Smoothing
Nomenclature for intact lipid molecule	Yes	Nomenclature for fragment ions	Yes

4) SE[M+NH4]+ / Lipid quantification

Quantitative	Yes	MS Level for quantification	MS^1

Internal lipid standard(s) MS^1

Internal standard	Endogenous subclass
SE 18:1-d7	SE

Type of quantification	Internal standard amount	Response correction	No
Type I isotope correction	Yes	Limit of quantification	Signal threshold
Normalization to reference	Yes	Lipid Quantification Software	Lipid Data Analyzer (LDA)
Batch correction	No		

5) Cer[M+HCOO]- / Lipid identification

Lipid class	Cer	MS Level for identification	MS^2
Identification level	sn Position	MS^2 adduct	[M+HCOO]-

Fragments for identification

Fragment name
-(+HCOO)
LCB(-H6NO)
FA1(-CH2O)

Isotope correction at MS^2	Type 2	MS^2 verified by standard	Yes
Background check at MS^2	No	Did you presume assumptions for identification?	No
Check on:	Isomeric overlap, Isobaric overlap	Limit of detection	Signal threshold
Lipid Identification Software	Lipid Data Analyzer (LDA)	Data manipulation	Smoothing
Nomenclature for intact lipid molecule	Yes	Nomenclature for fragment ions	Yes

5) Cer[M+HCOO]- / Lipid quantification

Quantitative	Yes	MS Level for quantification	MS^1

Internal lipid standard(s) MS^1

Internal standard	Endogenous subclass
CER d18:1-d7/15:0	CER

Type of quantification	Internal standard amount	Response correction	No

Type I isotope correction	Yes	Limit of quantification	Signal threshold
Normalization to reference	Yes	Lipid Quantification Software	Lipid Data Analyzer (LDA)
Batch correction	No		

6) SM[M+HCOO]- / Lipid identification

Lipid class	SM	MS Level for identification	MS^2
Identification level	sn Position	MS^2 adduct	[M+HCOO]-
Fragments for identification			

Fragment name
-(CH3+HCOO)
-FA1(+O)

Isotope correction at MS^2	Type 2	MS^2 verified by standard	Yes
Background check at MS^2	No	Did you presume assumptions for identification?	No
Check on:	Isomeric overlap, Isobaric overlap	Limit of detection	Signal threshold
Lipid Identification Software	Lipid Data Analyzer (LDA)	Data manipulation	Smoothing
Nomenclature for intact lipid molecule	Yes	Nomenclature for fragment ions	Yes

6) SM[M+HCOO]- / Lipid quantification

Quantitative	Yes	MS Level for quantification	MS^1
Internal lipid standard(s) MS^1			

Internal standard	Endogenous subclass
SM d18:1/18:1-d9	CER

Type of quantification	Internal standard amount	Response correction	No
Type I isotope correction	Yes	Limit of quantification	Signal threshold
Normalization to reference	Yes	Lipid Quantification Software	Lipid Data Analyzer (LDA)
Batch correction	No		

7) PC[M+HCOO]- / Lipid identification

Lipid class	PC	MS Level for identification	MS^2
Identification level	sn Position	MS^2 adduct	[M+HCOO]-
Fragments for identification			

Fragment name
-(CH3+HCOO)
-FA1(-H)-(CH3+HCOO)
-FA2(+HO)-(CH3+HCOO)

Isotope correction at MS^2	Type 2	MS^2 verified by standard	Yes
Background check at MS^2	No	Did you presume assumptions for identification?	No

Check on:	Isomeric overlap, Isobaric overlap	Limit of detection	Signal threshold
Lipid Identification Software	Lipid Data Analyzer (LDA)	Data manipulation	Smoothing
Nomenclature for intact lipid molecule	Yes	Nomenclature for fragment ions	Yes

7) PC[M+HCOO]- / Lipid quantification

Quantitative	Yes	MS Level for quantification	MS^1

Internal lipid standard(s) MS^1

Internal standard	Endogenous subclass
PC 15:0/18:1-d7	PC

Type of quantification	Internal standard amount	Response correction	No
Type I isotope correction	Yes	Limit of quantification	Signal threshold
Normalization to reference	Yes	Lipid Quantification Software	Lipid Data Analyzer (LDA)
Batch correction	No		

8) PC O[M+HCOO]- / Lipid identification

Lipid class	PC O	MS Level for identification	MS^2
Identification level	sn Position	MS^2 adduct	[M+HCOO]-

Fragments for identification

Fragment name
-(CH3+HCOO)
-FA2(-H)

Isotope correction at MS^2	Type 2	MS^2 verified by standard	Yes
Background check at MS^2	No	Did you presume assumptions for identification?	No
Check on:	Isomeric overlap, Isobaric overlap	Limit of detection	Signal threshold
Lipid Identification Software	Lipid Data Analyzer (LDA)	Data manipulation	Smoothing
Nomenclature for intact lipid molecule	Yes	Nomenclature for fragment ions	Yes

8) PC O[M+HCOO]- / Lipid quantification

Quantitative	Yes	MS Level for quantification	MS^1

Internal lipid standard(s) MS^1

Internal standard	Endogenous subclass
PC 15:0/18:1-d7	PC

Type of quantification	Internal standard amount	Response correction	No
Type I isotope correction	Yes	Limit of quantification	Signal threshold
Normalization to reference	Yes	Lipid Quantification Software	Lipid Data Analyzer (LDA)
Batch correction	No		

9) PC P[M+HCOO]- / Lipid identification

Lipid class	PC P	MS Level for identification	MS^2
Identification level	sn Position	MS^2 adduct	[M+HCOO]-

Fragments for identification

Fragment name
-(CH3+HCOO)
-FA2(-H)

Isotope correction at MS^2	Type 2	MS^2 verified by standard	Yes
Background check at MS^2	No	Did you presume assumptions for identification?	No
Check on:	Isomeric overlap, Isobaric overlap	Limit of detection	Signal threshold
Lipid Identification Software	Lipid Data Analyzer (LDA)	Data manipulation	Smoothing
Nomenclature for intact lipid molecule	Yes	Nomenclature for fragment ions	Yes

9) PC P[M+HCOO]- / Lipid quantification

Quantitative	Yes	MS Level for quantification	MS^1

Internal lipid standard(s) MS^1

Internal standard	Endogenous subclass
PC 15:0/18:1-d7	PC

Type of quantification	Internal standard amount	Response correction	No
Type I isotope correction	Yes	Limit of quantification	Signal threshold
Normalization to reference	Yes	Lipid Quantification Software	Lipid Data Analyzer (LDA)
Batch correction	No		

10) PE[M-H]- / Lipid identification

Lipid class	PE	MS Level for identification	MS^2
Identification level	sn Position	MS^2 adduct	[M-H]-

Fragments for identification

Fragment name
HG(PE,140)
HG(PE,196)
-FA1(-H)
-FA2(-H)

Isotope correction at MS^2	Type 2	MS^2 verified by standard	Yes
Background check at MS^2	No	Did you presume assumptions for identification?	No
Check on:	Isomeric overlap, Isobaric overlap	Limit of detection	Signal threshold
Lipid Identification Software	Lipid Data Analyzer (LDA)	Data manipulation	Smoothing
Nomenclature for intact lipid molecule	Yes	Nomenclature for fragment ions	Yes

10) PE[M-H]- / Lipid quantification

Quantitative	Yes	MS Level for quantification	MS^1

Internal lipid standard(s) MS^1

Internal standard	Endogenous subclass
PE 15:0/18:1-d7	PE

Type of quantification	Internal standard amount	Response correction	No
Type I isotope correction	Yes	Limit of quantification	Signal threshold
Normalization to reference	Yes	Lipid Quantification Software	Lipid Data Analyzer (LDA)
Batch correction	No		

11) PE P[M-H]- / Lipid identification

Lipid class	PE P	MS Level for identification	MS^2
Identification level	sn Position	MS^2 adduct	[M-H]-

Fragments for identification

Fragment name
HG(PE,196)
-FA2(-H)

Isotope correction at MS^2	Type 2	MS^2 verified by standard	Yes
Background check at MS^2	No	Did you presume assumptions for identification?	No
Check on:	Isomeric overlap, Isobaric overlap	Limit of detection	Signal threshold
Lipid Identification Software	Lipid Data Analyzer (LDA)	Data manipulation	Smoothing
Nomenclature for intact lipid molecule	Yes	Nomenclature for fragment ions	Yes

11) PE P[M-H]- / Lipid quantification

Quantitative	Yes	MS Level for quantification	MS^1

Internal lipid standard(s) MS^1

Internal standard	Endogenous subclass
PE 15:0/18:1-d7	PE

Type of quantification	Internal standard amount	Response correction	No
Type I isotope correction	Yes	Limit of quantification	Signal threshold
Normalization to reference	Yes	Lipid Quantification Software	Lipid Data Analyzer (LDA)
Batch correction	No		

12) PG[M-H]- / Lipid identification

Lipid class	PG	MS Level for identification	MS^2
Identification level	sn Position	MS^2 adduct	[M-H]-

Fragments for identification

Fragment name
GP(153)
-FA1(-H)
-FA2(-H)

Isotope correction at MS^2	Type 2	MS^2 verified by standard	Yes
Background check at MS^2	No	Did you presume assumptions for identification?	No
Check on:	Isomeric overlap, Isobaric overlap	Limit of detection	Signal threshold
Lipid Identification Software	Lipid Data Analyzer (LDA)	Data manipulation	Smoothing
Nomenclature for intact lipid molecule	Yes	Nomenclature for fragment ions	Yes

12) PG[M-H]- / Lipid quantification

Quantitative	Yes	MS Level for quantification	MS^1

Internal lipid standard(s) MS^1

Internal standard	Endogenous subclass
PG 15:0/18:1-d7	PG

Type of quantification	Internal standard amount	Response correction	No
Type I isotope correction	Yes	Limit of quantification	Signal threshold
Normalization to reference	Yes	Lipid Quantification Software	Lipid Data Analyzer (LDA)
Batch correction	No		

13) PI[M-H]- / Lipid identification

Lipid class	PI	MS Level for identification	MS^2
Identification level	sn Position	MS^2 adduct	[M-H]-

Fragments for identification

Fragment name
HG(PI,241)
-FA1(-H)
-FA2(-H)

Isotope correction at MS^2	Type 2	MS^2 verified by standard	Yes
Background check at MS^2	No	Did you presume assumptions for identification?	No
Check on:	Isomeric overlap, Isobaric overlap	Limit of detection	Signal threshold
Lipid Identification Software	Lipid Data Analyzer (LDA)	Data manipulation	Smoothing
Nomenclature for intact lipid molecule	Yes	Nomenclature for fragment ions	Yes

13) PI[M-H]- / Lipid quantification

Quantitative	Yes	MS Level for quantification	MS^1

Internal lipid standard(s) MS^1

Internal standard	Endogenous subclass

PI 15:0/18:1-d7	PI

Type of quantification	Internal standard amount	Response correction	No
Type I isotope correction	Yes	Limit of quantification	Signal threshold
Normalization to reference	Yes	Lipid Quantification Software	Lipid Data Analyzer (LDA)
Batch correction	No		

14) PS[M-H]- / Lipid identification

Lipid class	PS	MS Level for identification	MS^2
Identification level	sn Position	MS^2 adduct	[M-H]-

Fragments for identification

Fragment name
GP(153)
-FA1(-H)
-FA2(-H)

Isotope correction at MS^2	Type 2	MS^2 verified by standard	Yes
Background check at MS^2	No	Did you presume assumptions for identification?	No
Check on:	Isomeric overlap, Isobaric overlap	Limit of detection	Signal threshold
Lipid Identification Software	Lipid Data Analyzer (LDA)	Data manipulation	Smoothing
Nomenclature for intact lipid molecule	Yes	Nomenclature for fragment ions	Yes

14) PS[M-H]- / Lipid quantification

Quantitative	Yes	MS Level for quantification	MS^1

Internal lipid standard(s) MS^1

Internal standard	Endogenous subclass
PS 15:0/18:1-d7	PS

Type of quantification	Internal standard amount	Response correction	No
Type I isotope correction	Yes	Limit of quantification	Signal threshold
Normalization to reference	Yes	Lipid Quantification Software	Lipid Data Analyzer (LDA)
Batch correction	No		

15) LPC[M+HCOO]- / Lipid identification

Lipid class	LPC	MS Level for identification	MS^2
Identification level	sn Position	MS^2 adduct	[M+HCOO]-

Fragments for identification

Fragment name
HG(PC,224)
-FA1(-H)-(CH3+HCOO)

-(CH3+HCOO)

Isotope correction at MS^2	Type 2	MS^2 verified by standard	Yes
Background check at MS^2	No	Did you presume assumptions for identification?	No
Check on:	Isomeric overlap, Isobaric overlap	Limit of detection	Signal threshold
Lipid Identification Software	Lipid Data Analyzer (LDA)	Data manipulation	Smoothing
Nomenclature for intact lipid molecule	Yes	Nomenclature for fragment ions	Yes

15) LPC[M+HCOO]- / Lipid quantification

Quantitative	Yes	MS Level for quantification	MS^1

Internal lipid standard(s) MS^1

Internal standard	Endogenous subclass
LPC 18:1-d7	LPC

Type of quantification	Internal standard amount	Response correction	No
Type I isotope correction	Yes	Limit of quantification	Signal threshold
Normalization to reference	Yes	Lipid Quantification Software	Lipid Data Analyzer (LDA)
Batch correction	No		

16) LPE[M-H]- / Lipid identification

Lipid class	LPE	MS Level for identification	MS^2
Identification level	sn Position	MS^2 adduct	[M-H]-

Fragments for identification

Fragment name
HG(PE,140)
HG(PE,196)
-FA1(-H)

Isotope correction at MS^2	Type 2	MS^2 verified by standard	Yes
Background check at MS^2	No	Did you presume assumptions for identification?	No
Check on:	Isomeric overlap, Isobaric overlap	Limit of detection	Signal threshold
Lipid Identification Software	Lipid Data Analyzer (LDA)	Data manipulation	Smoothing
Nomenclature for intact lipid molecule	Yes	Nomenclature for fragment ions	Yes

16) LPE[M-H]- / Lipid quantification

Quantitative	Yes	MS Level for quantification	MS^1

Internal lipid standard(s) MS^1

Internal standard	Endogenous subclass
LPE 18:1-d7	LPE

Type of quantification	Internal standard amount	Response correction	No
Type I isotope correction	Yes	Limit of quantification	Signal threshold

Normalization to reference	Yes	Lipid Quantification Software	Lipid Data Analyzer (LDA)
Batch correction	No		

17) LPS[M-H]- / Lipid identification

Lipid class	LPS	MS Level for identification	MS^2
Identification level	sn Position	MS^2 adduct	[M-H]-
Fragments for identification			

Fragment name
GP(153)
-FA1(-H)

Isotope correction at MS^2	Type 2	MS^2 verified by standard	Yes
Background check at MS^2	No	Did you presume assumptions for identification?	No
Check on:	Isomeric overlap, Isobaric overlap	Limit of detection	Signal threshold
Lipid Identification Software	Lipid Data Analyzer (LDA)	Data manipulation	Smoothing
Nomenclature for intact lipid molecule	Yes	Nomenclature for fragment ions	Yes

17) LPS[M-H]- / Lipid quantification

Quantitative	Yes	MS Level for quantification	MS^1
Internal lipid standard(s) MS^1			

Internal standard	Endogenous subclass
PS 15:0/18:1-d7	PS

Type of quantification	Internal standard amount	Response correction	No
Type I isotope correction	Yes	Limit of quantification	Signal threshold
Normalization to reference	Yes	Lipid Quantification Software	Lipid Data Analyzer (LDA)
Batch correction	No		
